# Uso de la inteligencia artificial en el diagnóstico de alteraciones de la citología cervicouterina: estudio observacional en población universitaria

**DOI:** 10.7705/biomedica.7651

**Published:** 2025-12-10

**Authors:** José Said Manzano-Chaya, Tania Mendoza-Herrera, Ernesto García-Ayala

**Affiliations:** 1 Grupo de Investigación en Patología Estructural, Funcional y Clínica, Departamento de Patología, Universidad Industrial de Santander, Bucaramanga, Colombia Universidad Industrial de Santander Universidad Industrial de Santander Bucaramanga Colombia

**Keywords:** detección precoz del cáncer, inteligencia artificial, aprendizaje profundo, prueba de Papanicolaou, neoplasias del cuello uterino, humanos, Early detection of cancer, artificial intelligence, deep learning, Papanicolaou smear, uterine cervical neoplasms, female, humans

## Abstract

**Introducción.:**

La citología convencional (prueba de Papanicolaou) continúa siendo un pilar del tamizaje del cáncer cervicouterino en Colombia, pero su utilidad se ve opacada por una gran carga laboral y bajo rendimiento diagnóstico. El uso de la inteligencia artificial puede proveer una solución a este problema, sin embargo, no hay estudios que evalúen su utilidad en nuestra población.

**Objetivo.:**

Evaluar y comparar la capacidad discriminativa de cuatro modelos de inteligencia artificial para detectar anormalidades en la citología cervicouterina.

**Materiales y métodos.:**

Se obtuvieron 650 imágenes de células de citología cervicouterina convencional de una población universitaria del nororiente colombiano, las cuales fueron sometidas a evaluación diagnóstica por un patólogo experto. Mediante el análisis de imágenes digitales y aprendizaje profundo, se entrenaron cuatro modelos de inteligencia artificial (DenseNet, InceptionV3, MobileNet y VGG19) con los datos de una base de citología de acceso público, determinando la capacidad discriminativa de los modelos con su respectiva sensibilidad, especificidad y área bajo la curva.

**Resultados.:**

MobileNet tuvo la mejor capacidad discriminativa [área bajo la curva (AUC) de 0,97) con una especificidad del 0,99 y sensibilidad de 0,78 para la detección de alteraciones en la citología cervicouterina. Por otro lado, InceptionV3 tuvo un mejor desempeño en el tamizaje, con sensibilidad del 0,93, especificidad de 0,82 y área bajo la curva de 0,947.

**Conclusiones.:**

Nuestros resultados ilustran las ventajas y desventajas de diferentes modelos de inteligencia artificial y la forma como podrían ayudar a mejorar el rendimiento del tamizaje con citología convencional o, incluso, servir como método de tamizaje primario para descartar los casos negativos, lográndose un desempeño diagnóstico comparable con el de la lectura convencional.

## Abstract (en)

**Introduction.:**

Conventional cervical cytology (Pap smear) remains a primary method for cervical cancer screening in Colombia, despite limitations in diagnostic yield and heavy workload. The potential of artificial intelligence to address these challenges is yet to be evaluated in our population.

**Objective.:**

To evaluate and compare the discriminative ability of four artificial intelligencebased models for the detection of abnormalities in Pap smears.

**Materials and methods.:**

A total of 650 images of Pap smear cells were obtained from a university cohort in northeastern Colombia. These images were subjected to diagnostic evaluation by an expert pathologist. Four artificial intelligence models (DenseNet, InceptionV3, MobileNet, and VGG19) were trained using data from a publicly available Pap smear database with digital image analysis and deep learning. The discriminative ability of the models was determined by calculating their sensitivity, specificity, and area under the curve.

**Results.:**

MobileNet showed the highest discriminative ability (AUC = 0.97), with a specificity of 0.99 and sensitivity of 0.78 for the detection of altered cells in Pap smears. On the other hand, InceptionV3 had the best performance capabilities for screening, with a sensitivity of 0.93, specificity of 0.82, and AUC of 0.947.

**Conclusions.:**

The results of this study illustrate the advantages and disadvantages of different artificial intelligence models and how their application could help improve the diagnostic performance of manual reading in cervical cancer screening or even serve as a primary screening method to rule out negative cases, by achieving a diagnostic performance comparable to that of manual reading.

A nivel mundial, el cáncer de cuello uterino ocupa el cuarto lugar entre los cánceres de mujeres, con un estimado de 570.000 nuevos casos con 311.000 muertes por esta enfermedad en el 2018, tendencia que ha ido en ascenso según cifras de Globocan ^1^. Las proyecciones señalan que, si se mantienen las tendencias actuales, el número de muertes atribuibles a esta enfermedad en las Américas podría aumentar hasta en el 45 % en el 2030 ^2^. Cerca del 90 % de los nuevos casos y muertes en el 2020, tuvieron lugar en los países de ingresos bajos y medianos, y las tasas de mortalidad de Latinoamérica y el Caribe fueron tres veces más altas que en Norteamérica, lo cual evidencia las enormes desigualdades en salud ^2^, por lo que es necesario fortalecer los programas de tamizaje y diagnóstico temprano en nuestra población.

La citología cervicouterina convencional continúa siendo un pilar del tamizaje del cáncer de cuello uterino en Colombia, pero su utilidad se ve opacada por requerir de personal experto que revise manualmente cientos de células de forma individual, un proceso laborioso que lleva tiempo y está expuesto a errores humanos, lo cual se traduce en mayores demoras y costos para el sistema de salud.

El creciente avance de la patología digital y la investigación en inteligencia artificial plantean una posible solución a este problema. La inteligencia artificial es un campo de la informática que busca crear programas computacionales que simulan la forma en la que funciona la mente humana, al crear algoritmos capaces de aprender y tomar decisiones basadas en la información presentada, el cual ha surgido como una forma de solucionar problemas complejos que normalmente requerirían el uso de la inteligencia humana ^3^, con aplicaciones en campos como el reconocimiento de imágenes ^4^, el reconocimiento de voz ^5^, la traducción de lenguajes ^6^, el reconocimiento facial ^7^ y el diagnóstico médico ^8^.

La patología computacional basada en inteligencia artificial es una rama emergente de la patología digital, con múltiples estudios que han demostrado que los sistemas asistidos por ella tienen el potencial de clasificar diferentes enfermedades.

Campanella *et al*. validaron un algoritmo basado en aprendizaje profundo de alta capacidad para clasificar 44.732 imágenes de tres tipos de cáncer, incluyendo el cáncer de próstata, el carcinoma basocelular y las metástasis de cáncer de mama a los ganglios linfáticos axilares, y obtuvieron un área bajo la curva superior a 0,98 para todos los tipos de cáncer. Su aplicación clínica podría permitir que los patólogos excluyeran hasta el 75 % de las láminas de histología, manteniendo un 100 % de sensibilidad diagnóstica ^9^.

Korbar *et al*. desarrollaron múltiples algoritmos de aprendizaje profundo para clasificar un set de 2.074 imágenes de cinco tipos de pólipos colorrectales, incluyendo pólipos hiperplásicos, aserrados sésiles y tradicionales, tubulares y tubulovellosos ^10^. La sensibilidad diagnóstica obtenida en este estudio fue del 93 % (IC_95%_: 89,0 - 95,9 %). En este mismo campo de estudio, Bychkov *et al*. combinaron redes convolucionales neuronales para predecir el pronóstico médico de 420 pacientes con cáncer colorrectal, basados en el análisis de micromatrices de tejidos, y lograron un área bajo la curva de 0,69 (*hazard ratio* = 2,3; IC_95%_: 1,79 - 3,03), un resultado mejor que el logrado por un consenso de expertos en patología (*hazard ratio* = 1,67; IC_95%_: 1,28 - 2,19; AUC = 0,58) ^11^.

En un estudio multicéntrico en el que se evaluaron diferentes algoritmos de diagnóstico por inteligencia artificial para identificar metástasis a los ganglios linfáticos, el mejor algoritmo tuvo un área bajo la curva de 0,994, resultados similares a los logrados por un panel de once patólogos diferentes, con un área bajo la curva de hasta 0.998 ^12^. De forma similar, en un estudio multicéntrico en China con una población de más de 700.000 pacientes, se demostró una tasa de acuerdo diagnóstico del 94,7 % entre la lectura convencional y la lectura por inteligencia artificial de citologías de cuello uterino y, al usar la inteligencia artificial para asistir al diagnóstico citológico convencional, se demostró una mejoría en la sensibilidad para la detección de lesiones intraepiteliales cervicales del 5,8 %, con una ligera disminución en la especificidad diagnóstica ^13^.

En Latinoamérica, Diniz *et al*. evaluaron la aplicación de seis diferentes arquitecturas de aprendizaje profundo para clasificar imágenes de citología cervicouterina convencional ^14^. Posteriormente, combinaron las tres mejores, realizando un ensamblaje que logró una precisión de 0,96 y una especificidad de 0,97, superando a estudios similares ^15^, Isidoro DWA, Silva R, Silva AC, Lima SM, Paiva AC. Automatic classification of cervical cell patches based on non-geometric characteristics. Proceedings of the 15^th^ International Joint Conference on Computer Vision, Imaging and Computer Graphics Theory and Applications (VISIGRAPP 2020); 2020; Valletta, Malta).

Por otro lado, investigadores de la Universidad de Valparaíso (Chile) usaron imágenes de citología en base líquida para entrenar un modelo basado en ResNet-50, y lograron gran sensibilidad (0,981), especificidad (0,979) y precisión (0,980); sin embargo, al aplicar este modelo en imágenes de citología convencional, demostraron una disminución considerable del desempeño diagnóstico (sensibilidad 0,688, especificidad 0,762 y precisión 0,8735) ^16^. Estos resultados demuestran que para lograr un adecuado desempeño en citología convencional, se debe entrenar al modelo con un set de imágenes que haya sido diseñado mediante la citología convencional.

A pesar de las investigaciones realizadas en este campo, William *et al*. demostraron que siguen existiendo debilidades en las técnicas disponibles para clasificar las imágenes de células de citología convencional de forma automática mediante diferentes algoritmos de inteligencia artificial ^17^. Además, en su revisión de la literatura encontraron que muchos de los trabajos se centraban en la clasificación de las células individuales disponibles en bases de datos *online* de acceso público -como, por ejemplo, la base de datos Herlev-, las cuales habían sido aisladas mediante sistemas automáticos de segmentación y preprocesadas para lograr condiciones imagenológicas ideales, lo cual sacrifica la comparabilidad, dado que en la práctica diaria las citologías convencionales presentan frecuentemente artificios de procesamiento, dobleces y superposición de células, lo cual afecta la reproducibilidad y la validez externa de estos métodos.

Hasta la fecha, la gran mayoría de estudios en el campo de la inteligencia artificial se han centrado en el diseño de nuevos modelos, usando bases de datos de acceso público; sin embargo, pocos son los estudios que han evaluado su aplicación práctica en un entorno poblacional. Además, la gran cantidad de algoritmos de inteligencia artificial que se están desarrollando continuamente, dificulta el proceso de escoger el mejor para esta tarea; por lo tanto, diseñamos el presente estudio para evaluar y comparar la capacidad discriminativa de cuatro diferentes modelos diagnósticos basados en la inteligencia artificial para diagnosticar anormalidades en la citología cervicouterina en un programa de tamizaje de cáncer de cuello uterino del nororiente colombiano.

## Materiales y métodos

### Diseño del estudio y participantes

Desde el 1° de enero hasta el 31 de diciembre del 2023, se condujo un estudio observacional analítico en el programa de tamizaje de cáncer de cuello uterino de un centro de salud de primer nivel del nororiente colombiano ubicado en la Universidad Industrial de Santander, el cual ofrece servicios de promoción y prevención de sus distintos programas de pregrado y posgrado a los estudiantes.

Se obtuvieron datos clínicos relevantes junto con un extendido de citología cervicouterina con coloración de Papanicolaou por cada participante, y se tomaron fotografías digitales de las células representativas de cada caso con un microscopio óptico de luz de marca Leica DM750™ conectado a una cámara digital con referencia ICC50W y resolución de 5,0 megapíxeles, usando un aumento de 40X.

### Uso de la inteligencia artificial

Uno de los pasos más importantes a la hora de diseñar un estudio de inteligencia artificial en patología, es escoger una base de datos con el suficiente número de casos para que sirva como set de entrenamiento ^18^. Debido a que no hay estudios en Colombia sobre el uso de la inteligencia artificial para evaluar muestras de citología cervicouterina, se escogió una base de datos derivada del estudio de Diniz *et al*. ^14^, quienes evaluaron el rendimiento, la capacidad discriminativa y la validez externa, con pacientes latinoamericanas. Esta base de datos contiene imágenes de células previamente balanceadas y clasificadas en dos, tres y seis categorías diagnósticas diferentes, tomando imágenes de la base de datos del *Center for Recognition and Inspection of Cells* (CRIC) disponible en línea en: https://database.cric.com.br ^19^.

Para el set de entrenamiento del presente estudio, usamos el de dos categorías, que contienen casi 7.000 imágenes de células procesadas independientemente de forma manual, seleccionando el centro del núcleo y cortando un área de 90 x 90 píxeles a su alrededor. Esta metodología permite evaluar cada célula independientemente, incluyendo el núcleo y parte del citoplasma en la imagen final, y evita la aparición de dos o más núcleos en la misma imagen. El siguiente paso consistió en usar esta metodología en la muestra estudiada, con lo cual se obtuvo un total de 650 células individuales.

**Figura 1:**
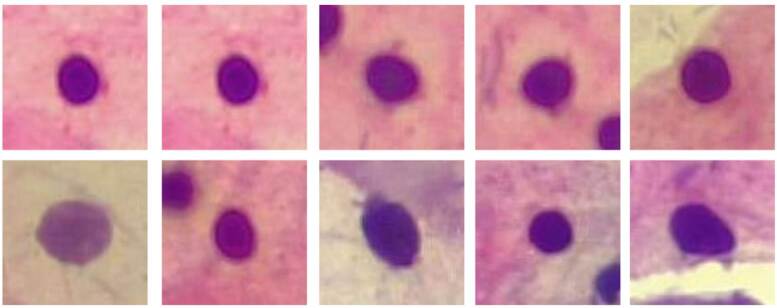
Ejemplo de las células clasificadas como normales por el patólogo experto

**Figura 2:**
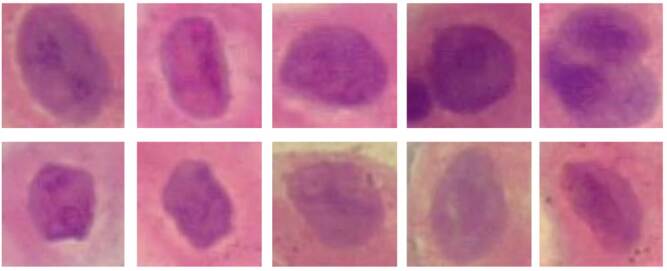
Ejemplo de las células clasificadas como alteradas por el patólogo experto

Posteriormente, las imágenes de la muestra fueron clasificadas por un patólogo experto en dos grupos: uno de células normales (sin alteraciones morfológicas) y otro que incluyó todas aquellas que presentaran alteraciones asociadas con lesiones premalignas o malignas, según los criterios del sistema Bethesda ^20^. En la figura 1 se presenta un ejemplo de las células clasificadas como normales y, en la figura 2, uno de las células clasificadas como anormales.

A continuación, las imágenes de las células correspondientes al set de entrenamiento se cargaron en un *software* de inteligencia artificial desarrollado por Krãter *et al*. ^21^ -del *Center for Molecular and Cellular Bioengineering* (CMCB) de Dresde (Alemania)- llamado “AIDeveloper”, el cual permite entrenar y evaluar diferentes modelos de inteligencia artificial con una interfaz que facilita al patólogo interactuar con el *software*.

Con el fin de comparar la capacidad discriminativa entre diferentes inteligencias artificiales y su rendimiento tomando como referencia el diagnóstico del médico patólogo experto, decidimos usar los siguientes modelos de inteligencia artificial cuya arquitectura se basa en el aprendizaje profundo: DenseNet ^22^, Inception V3 ^23^, Szegedy C, Vanhoucke V, Ioffe S, Shlens J, Wojna Z. Rethinking the inception architecture for computer vision. Proceedings of the IEEE Conference on Computer Vision and Pattern Recognition; 2016 Jun 27-30; Las Vegas, NV, USA. p. 2818-26. 10.1109/ CVPR.2016.308), MobileNet ^24^ y VGG19 ^25^.

Estos modelos de inteligencia artificial habían sido previamente entrenados con más de un millón de imágenes de la base de datos ImageNet. Esto confiere la ventaja de poder hacer *transfer-learning* en nuestro estudio, un proceso que permite mejorar el desempeño y la velocidad de aprendizaje de distintos modelos de inteligencia artificial al crear una relación lógica entre las bases de datos a las que ha sido previamente expuesto el modelo y la base de datos actual ^26^. No obstante, debido a que las imágenes son tomadas de fotografías de entornos naturales (como carreteras, casas o personas caminando), tienden a mantener su verticalidad con respecto al eje horizontal, lo que puede causar que los modelos preentrenados de esta forma sobreestimen la verticalidad de las imágenes a las que sean expuestos (18, Mikolajczyk A, Grochowski M. Data augmentation for improving deep learning in image classification problem. In: 2018 International Interdisciplinary PhD Workshop (IIPHDW); 2018. https://doi.org/10.1109/IIPHDW.2018.8388338).

Con el fin de evitar este fenómeno, las imágenes de la base de datos de entrenamiento se transformaron agregando ruido estadístico, modificando ligeramente su enfoque y rotándolas de forma aleatoria. Cada una de las transformaciones elegidas fueron usadas cada dos ciclos de entrenamiento logarítmico del conjunto de datos (*epoch*).

**Figura 3:**
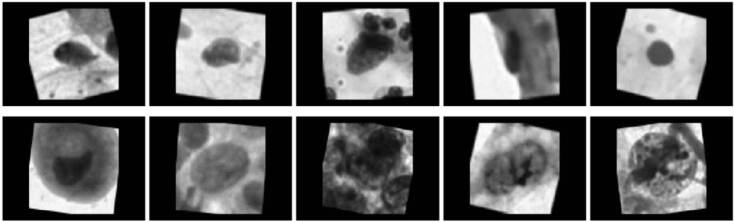
Ejemplos de imágenes de células normales (fila superior) y con alteraciones (fila inferior) de la base de datos usada para entrenar los modelos de la inteligencia artificial, después de las transformaciones

Además, las imágenes fueron convertidas a escala de grises, dado que, por experiencia de los investigadores en el campo, este paso permite mejorar considerablemente el desempeño del modelo, probablemente al disminuir el ruido estadístico que causan diferentes colores dentro de una imagen de citología. En la figura 3, se muestran ejemplos de las imágenes de células usadas para entrenar los modelos después de haber hecho las modificaciones.

Finalmente, cada uno de los modelos de inteligencia artificial se entrenó de forma supervisada usando el 80 % de las imágenes de la base de datos de referencia como set de entrenamiento y, el 20 % restante, como set de validación, hasta lograr la mejor capacidad discriminativa posible, para clasificar posteriormente cada una de las células de nuestra muestra.

Todo el proceso se llevó a cabo en un computador portátil con 16 GB de memoria RAM, procesador Core i7-9750H, tarjeta gráfica NVIDIA GeForce GTX 1660Ti de 6 GB y sistema operativo Windows 10. Para cuantificar y comparar los datos obtenidos, se calcularon la sensibilidad, la especificidad, el valor predictivo positivo, el valor predictivo negativo y el área bajo la curva (AUC-ROC) de cada uno de los modelos de inteligencia artificial.

### Consideraciones éticas

Todas las participantes fueron informadas sobre el objetivo del estudio y firmaron un consentimiento informado aceptando su participación en el mismo. El protocolo del estudio había sido revisado y aprobado previamente por el Comité de Ética de la Universidad Industrial de Santander.

## Resultados

### Datos clínicos

Desde el 1° de enero hasta el 31 de diciembre del 2023, se incluyeron 65 muestras de citología cervicouterina de mujeres entre los 18 y los 29 años de edad (promedio de 21 años), con sus respectivos reportes. En la caracterización demográfica, el 96,9 % provenía de la zona urbana. Hubo 41 (63 %) participantes que informaron usar algún tipo de método anticonceptivo; entre ellas, los más usados fueron las pastillas anticonceptivas y las pastillas anovulatorias (25 casos, 60 %), los métodos de barrera (14 casos, 34 %) y el dispositivo intrauterino (2 casos, 4,8 %). El 51 % de las 65 participantes reportaron haberse practicado una citología cervicouterina previa y solo en el 15 % de ellas hubo hallazgos anormales en los resultados de dicho examen.

De las 65 pacientes, 49 (75 %) informaron haber recibido la vacuna contra el virus del papiloma humano (HPV), incluida en el programa ampliado de inmunización del Ministerio de Salud y Protección Social de Colombia desde el 2013, cuya aplicación consiste en tres dosis con intervalos de 0, 6 y 60 meses ^27^. Se pudo concluir que existe una falencia sobre la cobertura, pues en el presente estudio, solo 7 (14 %) de las pacientes habían recibido el esquema completo de tres dosis, 25 (51 %) recibieron dos dosis y, 12 (24,4 %), solo una dosis.

Todas las 65 muestras recibidas contaron con una calidad satisfactoria de acuerdo con los criterios del sistema Bethesda ^20^. De las 650 células individuales de la muestra total, 455 (70 %) fueron clasificadas por el patólogo experto como células normales, y las 195 (30 %) restantes se clasificaron dentro del grupo de atipia citológica. Entre estas, 82 (42 %) correspondieron a una lesión intraepitelial escamosa de bajo grado y 113 (58 %) correspondieron a anormalidades de células escamosas de significado indeterminado (ASCUS), según los criterios del sistema Bethesda ^20^. No hubo células con lesiones de alto grado, ni anormalidades epiteliales glandulares.

### Resultados de inteligencia artificial

#### Desempeño con la base de datos de referencia

La base de datos de referencia se dividió en un set de entrenamiento (80 % de los datos) y otro de validación (el 20 % restante). Los datos reportados corresponden al desempeño de cada uno de los modelos de inteligencia artificial con el set de validación, tras finalizar los ciclos de entrenamiento con la base de datos de referencia (cuadro 1). Estos resultados resaltan la validez del entrenamiento de los modelos, los cuales se compararon posteriormente según el desempeño logrado con la base de datos de las pacientes.

**Cuadro 1:** Desempeño de los cuatro modelos de inteligencia artificial con la base de datos de referencia

Modelo	Sensibilidad	Especificidad	VPP	VPN	AUC (curva ROC)
DenseNet	0.94	0,90	0,90	0,95	
InceptionV3	0.96	0,89	0,88	0,96	0,98
VGG19	0.95	0,84	0,82	0,96	0,96
MobileNet	0.92	0,92	0,92	0,92	0,97

La mejor sensibilidad se obtuvo con InceptionV3, (0,96), seguida por la de VGG19, DenseNet y MobileNet (0,95, 0,94 y 0,92, respectivamente); y el mejor desempeño en especificidad se obtuvo con MobileNet, de 0,92. En general, todos los modelos contaron con un excelente valor de área bajo la curva (0,96 - 0,98), lo que resalta su capacidad como clasificadores binarios para diferenciar células normales y células con alteraciones.

**Figura 4:**
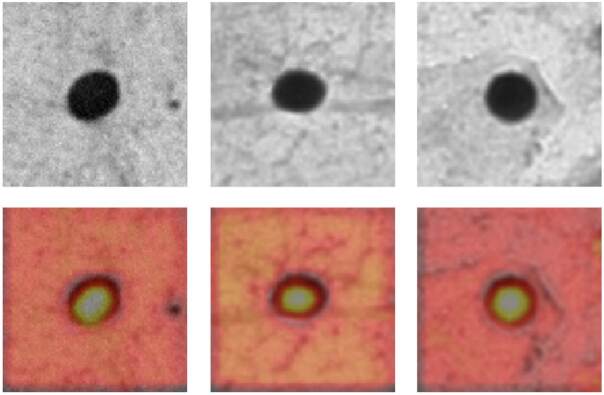
Ejemplo de células normales clasificadas correctamente por DenseNet

**Figura 5:**
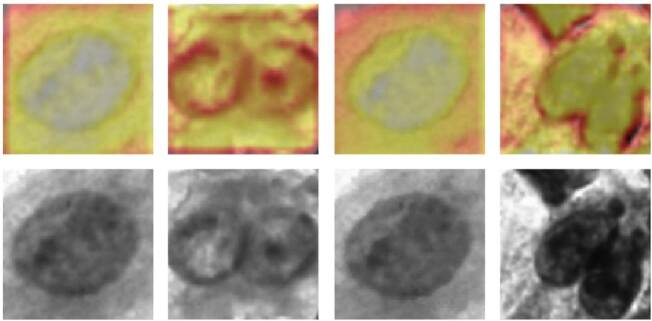
Ejemplos de células con alteraciones clasificadas correctamente por DenseNet

Debido a que solo vemos el resultado del entrenamiento, es difícil entender cómo los modelos de inteligencia artificial llegaron a tomar las decisiones necesarias para clasificar las células como normales o alteradas, y si esos métodos fueron similares o diferentes a los que usan los patólogos. Una forma de lograr entender este proceso es mediante el uso de mapas de activación de clase ponderada por gradiente (*Grad-CAM*), los cuales resaltan las regiones más importantes de la imagen para su clasificación mediante un gradiente de colores (Korbar B, Olofson AM, Miraflor AP, Nicka CM, Suriawinata MA, Torresani L, *et al*. Looking under the hood: Deep neural network visualization to interpret whole-slide image analysis outcomes for colorectal polyps. Proceedings of the IEEE Conference on Computer Vision and Pattern Recognition (CVPR); 2017. p. 821-7). En la figura 4 se muestra un ejemplo de células normales clasificadas como normales y, en la figura 5, uno de células con alteraciones citológicas clasificadas adecuadamente por DenseNet.

Estas gráficas se crean usando un gradiente de colores que va desde negro y rojo para las áreas menos importantes, hasta amarillo y blanco para las áreas más relevantes. Al comparar estas dos figuras, se puede observar que el tamaño y la forma del núcleo cobran una gran importancia a la hora de clasificar una célula. En la figura 4, la mayor parte de la activación está centrada en el núcleo y en su contorno que, al ser redondeado, pequeño y regular, lleva a clasificar esta célula como normal. En la figura 5, se puede observar una activación más heterogénea, con puntos de mayor relevancia en la clasificación, centrados en alteraciones de la cromatina nuclear, el contorno nuclear y el citoplasma. Por tanto, se puede concluir que las variaciones en la densidad de la cromatina, las alteraciones en el contorno nuclear y la menor cantidad de citoplasma (que podrían traducirse en alteraciones en la relación núcleo-citoplasma), llevan a clasificar estas células como alteradas. Estos parámetros están en concordancia con los usados por los patólogos para diagnosticar anormalidades en la citología cervicouterina, estandarizados por el sistema Bethesda ^20^.

### Desempeño con la base de datos de prueba

Tras finalizar el entrenamiento de los modelos y verificar que tuvieran un adecuado desempeño con la base de datos de referencia, probamos su desempeño en la base de datos de la población de estudio. Todas las células se usaron como set de prueba y los resultados obtenidos se muestran en el cuadro 2. Además, la matriz de confusión de cada modelo se muestra en el cuadro 3.

**Cuadro 2:** Desempeño de los cuatro modelos de inteligencia artificial con la base de datos de prueba

Modelo	Sensibilidad	Especificidad	VPP	VPN	AUC (curva ROC)
DenseNet	0,81	0,96	0,90	0,92	0,948
InceptionV3	0,93	0,82	0,68	0,97	0,947
VGG19	0,83	0,96	0,90	0,93	0,948
MobileNet	0,78	0,99	0,96	0,91	0,967

**Cuadro 3:** Matriz de confusión de los cuatro modelos de inteligencia artificial con la base de datos de prueba, comparados con el método de referencia

Modelo de inteligencia artificial		Método de referencia	
		Alteración	Normal
DenseNet	Alteración Normal	158	17
		37	438
InceptionV3	Alteración Normal	182	84
		13	371
VGG19	Alteración Normal	161	17
		34	438
MobileNet	Alteración Normal	153	6
		42	449

Entre los cuatro modelos de inteligencia artificial usados, el que obtuvo la mejor área bajo la curva fue MobileNet, el cual se destacó también por su gran especificidad, a costa de una sensibilidad inferior. Debido a que la prueba de citología cervicouterina se usa para el tamizaje diagnóstico, la prueba elegida debería tener la mejor sensibilidad posible, con el fin de poder detectar la mayor cantidad de pacientes con resultados positivos. Para este propósito, el mejor modelo de inteligencia artificial fue InceptionV3, el cual mostró la mejor sensibilidad, tanto en el set de entrenamiento como en el set de prueba, a costa de una especificidad y de un valor predictivo positivo menor que otros modelos.

## Discusión

En Colombia, el plan de beneficios de salud incluye la citología cervicouterina como una de las tres pruebas de tamizaje obligatorias para el cáncer de cuello uterino, especialmente, en la población joven de 25 a 29 años, junto con el test de ADN-HPV y las técnicas de inspección visual con ácido acético y lugol ^27^. La lectura convencional morfológica basada en los criterios del sistema Bethesda es el método diagnóstico de referencia en citología cervicouterina; sin embargo, su sensibilidad reportada en grandes series de casos varía entre el 30 y el 87 %, y su especificidad, entre el 86 y el 100 % ^28^. En comparación, InceptionV3 mostró una sensibilidad mayor que la lectura convencional reportada en la literatura (0,93), a costa de una especificidad ligeramente menor (0,82). Por otro lado, DenseNet, VGG19 y MobileNet mostraron una sensibilidad de 0,81, 0,83 y 0,78, con especificidad de 0,96, 0,96 y 0,99, respectivamente, valores comparables con los de la lectura convencional de muestras trabajadas manualmente por patólogos expertos ^28^^,^^29^.

En la literatura disponible sobre este tema, S. Sornapudi *et al*. ^30^ compararon el uso de DenseNet, VGG19 e InceptionV3 en imágenes de citología en base líquida, y obtuvieron valores de sensibilidad de 0,80, 0,89 y 0,91, y valores de área bajo la curva de 0,94, 0,95 y 0,88, respectivamente. Estos resultados son similares a los obtenidos en el presente estudio con el set de prueba, resaltándose que tanto la sensibilidad como el área bajo la curva de InceptionV3 fueron superiores en ambas bases de datos.

En este trabajo, la toma de fotografías y la segmentación celular se hicieron de forma manual, un paso que toma tiempo y hace que este sistema sea menos práctico para su uso diario. Para solventar este problema, diferentes autores han desarrollado dos metodologías principales. La primera es escanear toda la lámina (*whole-slide imaging*, WSI) para, posteriormente, separar y clasificar las células mediante inteligencia artificial. Esta metodología es la más frecuente en otros estudios ^31^ y fue usada por Bao *et al*. ^13^ en un estudio de cohorte a gran escala, que incluyó 0.,7 millones de mujeres en China, en el cual lograron mejorar la sensibilidad diagnóstica, con un índice de acuerdo del 99 % entre el diagnóstico por lectura de muestras trabajadas manualmente y el de aquellas leídas mediante inteligencia artificial.

El segundo método consiste en hacer uso de la realidad aumentada con clasificación de células por inteligencia artificial en tiempo real para asistir en la lectura de muestras preparadas manualmente. Esta metodología tiene la ventaja de poder usarse fácilmente en la práctica diaria; además, en un estudio conducido por Tang *et al*., se demostró que podía aumentar la sensibilidad y la tasa de acuerdo interobservador en la citología cervicouterina, con un modelo de inteligencia artificial cuya sensibilidad fue del 0,90 y un área bajo la curva de 0,81, valores ligeramente inferiores a los obtenidos en el presente estudio ^32^.

Debido a que este estudio fue conducido en un programa de tamizaje de población joven, los resultados de la muestra incluyeron solo lesiones de bajo grado y ASCUS, por lo cual se tuvo que limitar la evaluación de inteligencia artificial a la clasificación de dos categorías (normales o alteradas). Se considera que futuros estudios sobre este tema podrían incluir una población más amplia para evaluar el desempeño de estos modelos en lesiones de alto grado (ASC-H, HSIL) y carcinoma *in situ*.

En el conocimiento de los autores, este es el primer estudio desarrollado en Colombia que investiga la aplicabilidad práctica de la inteligencia artificial en el tamizaje de cáncer de cuello uterino. En este trabajo, se usó *transfer-learning* y técnicas de transformación de imágenes para disminuir el *over-fitting* y mejorar el desempeño diagnóstico de diferentes modelos de inteligencia artificial; usamos Grad-CAM para entender los criterios que usa la inteligencia artificial a la hora de clasificar células y cómo se comparan con los criterios usados por citopatólogos expertos. Posteriormente, se evaluaron las ventajas y desventajas de cada uno para clasificar imágenes de células en la citología cervicouterina convencional. Los resultados son comparables con los de la lectura convencional ^28^^,^^29^ y, similares o superiores en sensibilidad y área bajo la curva, con respecto a estudios similares publicados sobre este tema ^30^^,^^32^.

Es importante mencionar que, debido al número limitado de participantes, el set de datos de pacientes colombianas fue menor que el ideal para el diseño de esta investigación. Esto representa una limitación a la hora de sacar conclusiones y disminuye la validez externa del presente estudio. Sin embargo, se considera que los resultados representan el primer paso hacia construir un sistema de clasificación que permita ayudar en el proceso del diagnóstico en citología cervicouterina. La evidencia científica actual señala que el desarrollo de un sistema basado en inteligencia artificial podría mejorar la sensibilidad y el tiempo de oportunidad de reporte de citología cervicouterina ^33^; incluso, podría llegar a servir como un método de tamizaje primario para descartar casos negativos, disminuyendo así la carga de trabajo diaria del patólogo, sin alterar el rendimiento diagnóstico ^13^. Sin embargo, se requieren estudios adicionales para evaluar esta posibilidad en nuestra población.
